# A case of adenosquamous pancreatic cancer with a KRAS G12C mutation with an exceptional response to immunotherapy

**DOI:** 10.18632/oncotarget.28659

**Published:** 2024-10-11

**Authors:** Murtaza Ahmed, Brent K. Larson, Arsen Osipov, Nilofer Azad, Andrew Hendifar

**Affiliations:** ^1^Department of Internal Medicine, Cedars Sinai Medical Center, Los Angeles, CA 90048, USA; ^2^Department of Pathology and Laboratory Medicine, Cedars Sinai Medical Center, Los Angeles, CA 90048, USA; ^3^Department of Internal Medicine (Division of Hematology-Oncology) Cedars Sinai Medical Center, Los Angeles, CA 90048, USA; ^4^Department of Oncology, Sidney Kimmel Comprehensive Cancer Center, Johns Hopkins University, Baltimore, MD 21218, USA

**Keywords:** pancreatic cancer, immunotherapy, metastasis

## Abstract

Pancreatic ductal adenocarcinoma (PDAC) is a leading cause of cancer-related deaths, with adenosquamous carcinoma of the pancreas (ASCP), a rare variant, representing 1–10% of cases. Standard chemotherapy trials for pancreatic cancer exclude ASCP, leaving its optimal treatment uncertain. This report describes a 68-year-old male with metastatic ASCP and a KRAS G12C mutation, progressing through multiple lines of systemic therapy, including targeted inhibition of KRAS G12C. Notably, the patient exhibited a robust response to single-agent immune checkpoint inhibition (ICI) with pembrolizumab, despite intact mismatch repair proteins. The limited success of traditional therapies in pancreatic cancer, coupled with the rarity of ASCP, presents a challenge in establishing effective treatment strategies. While KRAS G12C inhibitors demonstrated modest benefits, this case highlights the remarkable response to ICI in a patient with squamous histology. The distinct tumor microenvironment of ASCP, characterized by squamous differentiation, may contribute to this exceptional response. This underscores the need for further research and clinical trials focused on ICI in ASCP, with an ongoing multi-center phase 2 trial investigating outcomes in this specific subset.

## INTRODUCTION

Pancreatic cancer is the third most common cause of cancer death in the United States, responsible for a projected 50,555 deaths in 2023 [1]. The incidence of pancreatic cancer continues to increase every year, and mortality has increased by 5% since 2000 [1]. Pancreatic ductal adenocarcinoma (PDAC) represents approximately 90% of all pancreatic cancers, whereas adenosquamous carcinoma of the pancreas (ASCP), a rare and aggressive variant of PDAC defined by at least 30% squamous differentiation, represents 1–10% of cases [2]. Given the lack of validated screening or early symptoms, 80–90% of patients initially present with stage III or IV cancer, at which tumors are unresectable. For these patients the only option is systemic therapy, with a median overall survival (OS) ranging from 6.9 to 9.3 months [3]. Importantly, the trials that have established the present standard of care for chemotherapy in pancreatic cancer only enrolled pancreatic adenocarcinoma, so the true benefit of systemic chemotherapy in ASCP is wholly unknown.

Here, we present a case of metastatic ASCP with a *KRAS* G12C mutation refractory to multiple lines of systemic therapy, including platinum-based chemotherapy and targeted inhibition of *KRAS* G12C. Subsequently, the cancer demonstrated an excellent response to single agent immune checkpoint inhibition.

## CASE PRESENTATION

A 68-year-old man presented to our institution for recommendations regarding a diagnosis of advanced pancreatic cancer with progression on standard chemotherapy. Importantly, he provided informed written consent for the gathering and publication of his clinical, genomic and molecular data as well as deidentified images. He initially presented at an outside hospital in March 2019 with worsening abdominal pain. History revealed no relevant family or prior smoking history. Abdominal CT scan and subsequent PET-CT revealed a pancreatic head mass and multiple liver lesions. In June 2019, two core biopsies of a liver lesion revealed poorly differentiated carcinoma. Subsequently, in November 2019, a fine-needle aspiration (FNA) of the pancreatic mass demonstrated poorly differentiated carcinoma with glandular features. Given an ECOG performance status of 1, the patient began systemic therapy for presumed PDAC with FOLFIRINOX (leucovorin, 5-fluorouracil, irinotecan, oxaliplatin), with an initial radiographic response. He subsequently had other systemic regimens over a 2-year period without a significant response and with progressive abdominal pain. This prompted him to present to our institution for further evaluation and care.

**Figure 1:**
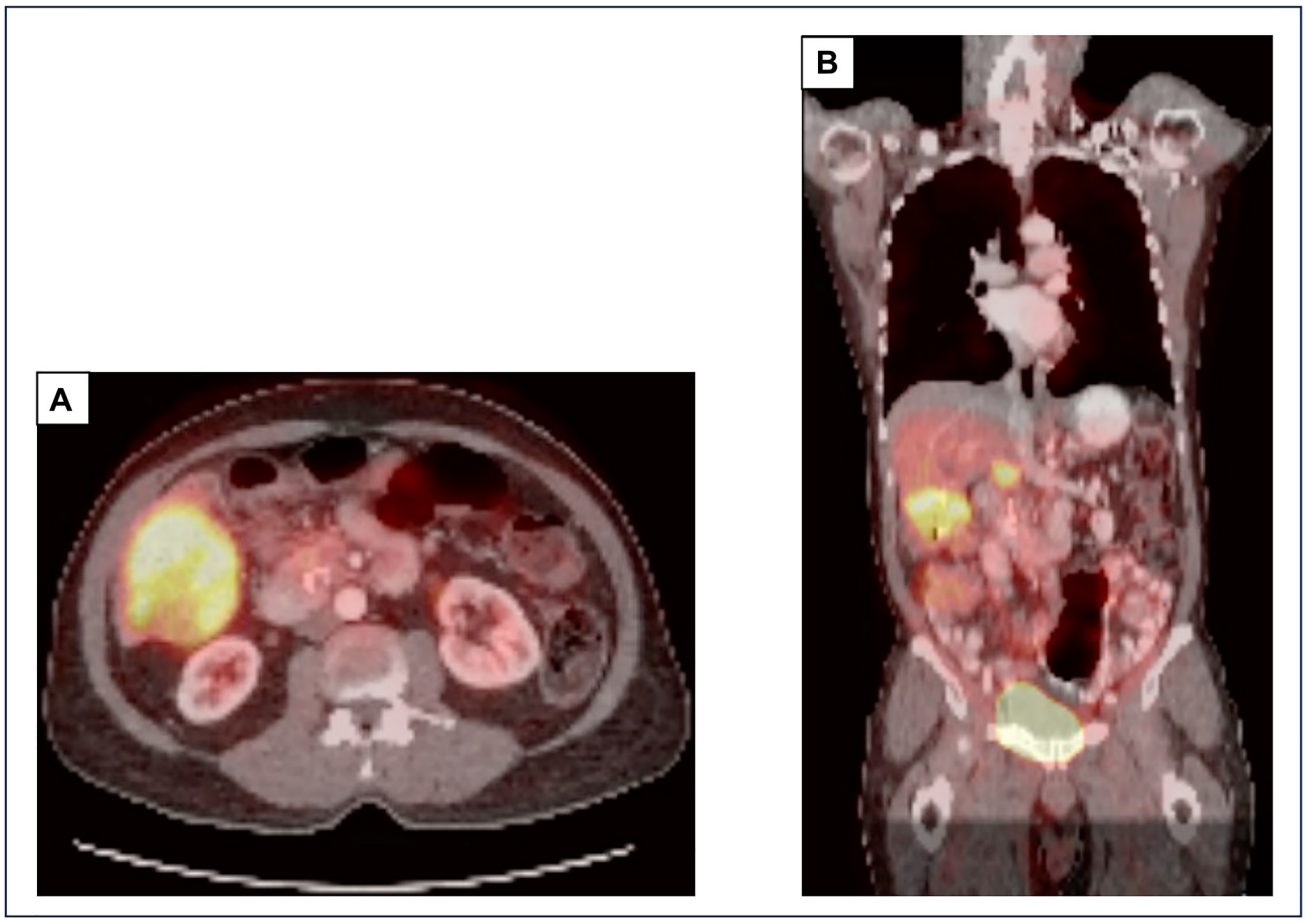
PET-CT scan at our institution prior to staring sotorasib demonstrates an 8.2 cm mass in the right inferior lobe of the liver with a SUV of 10.8 (**A**, **B**) and a 2.7 cm pancreatic head mass with a SUV of 5.8 (B). Abbreviations: PET: positron emission tomography; CT: computed tomography.

**Figure 2:**
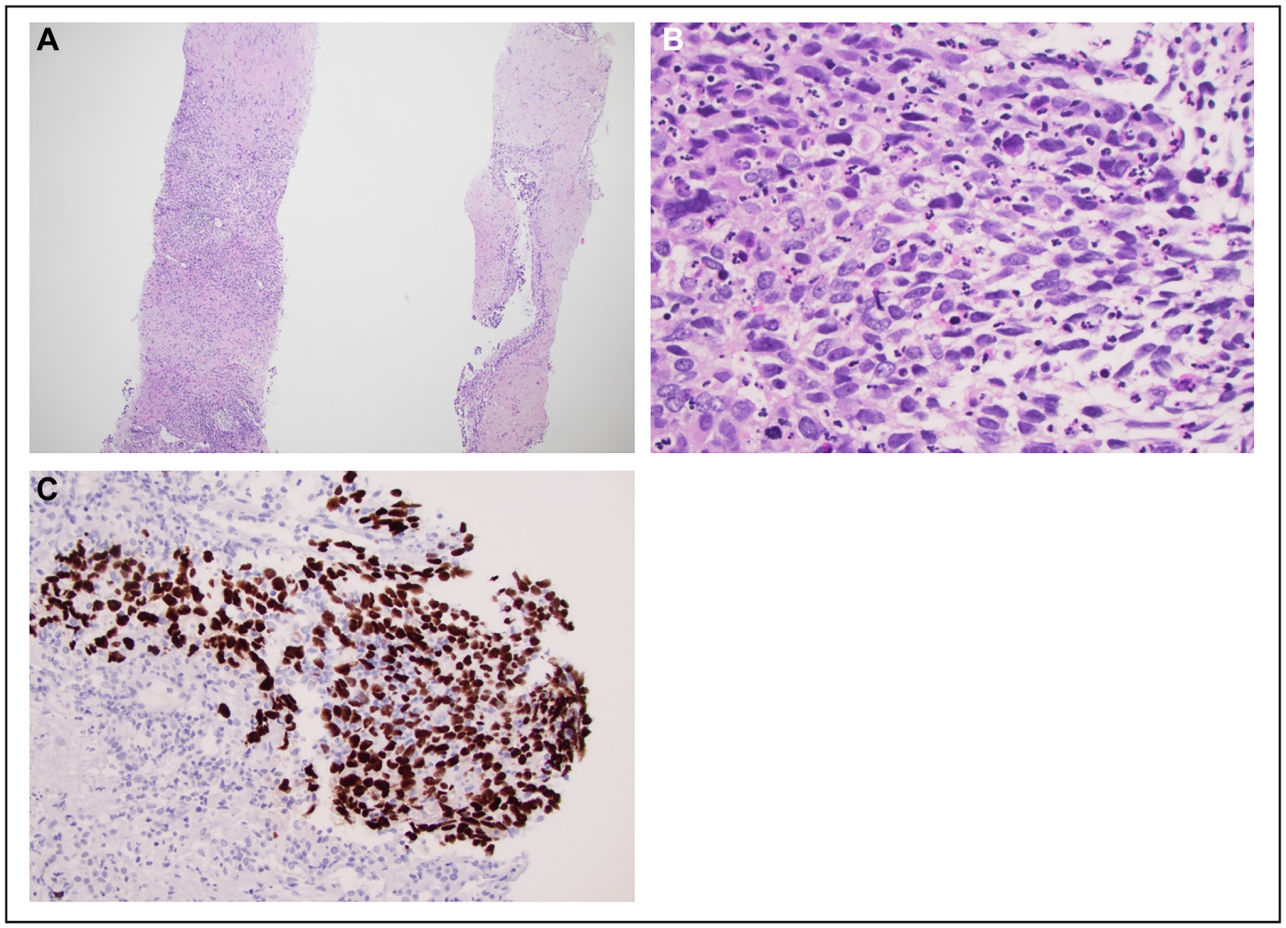
Histopathological and imrnunohistochemical images of a core biopsy of the liver lesion. (**A**) Low-power view of the liver biopsy demonstrating dense fibrosis and patchy inflammation with infiltration by nests of atypical cells (hematoxylin and eosin (H&E) stain, 40x magnification). (**B**) High-power examination of the tumor cells demonstrates nests of atypical cells with basaloid morphology, including elongated and enlarged nuclei, hyperchromasia, and high nucleus-to-cytoplasm ratios (H&E stain, 400x magnification). (**C**) Immunostaining for p40 demonstrates diffuse positive staining in tumor cell nuclei, supportive of squamous differentiation (p40 immunostain, 200x magnification).

**Figure 3:**
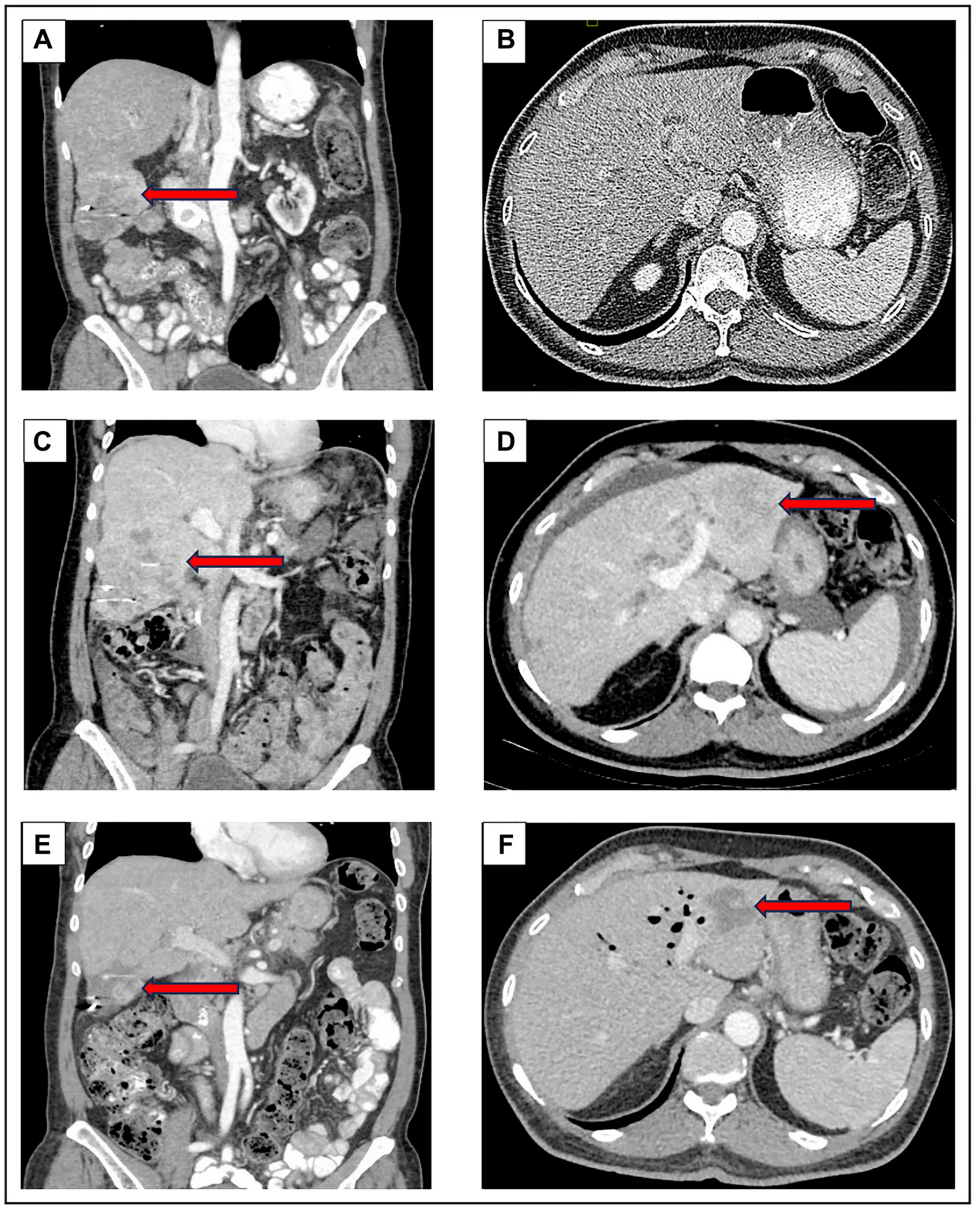
CT Scan on initial presentation to our institution in October 2022 (**A**, **B**) demonstrated a right lobe liver lesion measuring 8.2 cm in greatest dimension. In April 2023 (**C**, **D**), after six months of treatment with sotorasib, there was an increase in the right lobe liver lesion to 10.2 cm along with a new left lobe lesion measuring 5 cm. In July 2023 (**E**, **F**), after four cycles of pembrolizumab, the right lobe liver lesion reduced in size to 5.7 cm and the left liver lobe lesion decreased to 3.5 cm.

At our institution, the patient underwent a PET-CT scan, revealing that all the measurable lesions were FDG- avid: an 8.2 cm mass in the right inferior hepatic lobe with standard uptake value (SUV) of 10.8 and a 2.7 cm pancreatic head mass with SUV of 5.8 (Figure 1). A pathology review at our institution of the two prior liver core biopsies revealed a poorly differentiated carcinoma with squamous differentiation, as indicated by diffuse positive staining for p40, a recognized marker for squamous cells. (Figure 2). Next-generation sequencing (NGS) via the Oncomine^™^ Comprehensive Assay Plus panel identified pathogenic mutations in KRAS, ARID1A, TP53, and KDM6A (Table 1), along with a low tumor mutational burden (TMB) of 5 mutations per megabase (mut/Mb) and indeterminate microsatellite instability (MSI-I). The programmed death ligand-1 (PD-L1) and mismatch repair (MMR) protein status could not be assessed due to limited tissue availability, however records from the outside institution demonstrate intact MMR proteins via immunohistochemistry. These findings, combined with the pathology report from the previous FNA and exclusion of an extrapancreatic malignancy, led our tumor board to conclude that the neoplasm was likely an ASCP, with the adenocarcinoma component unsampled in the liver. Considering the presence of the *KRAS* G12C mutation, he was started on sotorasib in November 2022, which he tolerated well. Follow-up imaging in December 2022 and February 2023 indicated stable disease. However, in April 2023, a CT scan revealed an increase in size of the right hepatic metastatic lesion and a new 5.0 cm lesion in the left lateral hepatic lobe (Figure 3).

**Table 1:** Genetic mutations detected in next-generation sequencing

Gene	Chromosome position	Nucleotide change	Amino acid change	Molecular consequence	Allele frequency (%)
**KRAS**	12: 25398285	c.34G> T	p.G12C	Missense	19.0
**KDM6A**	X: 27094421	c.3129dupT	p.D589Rfs*8	Frameshift	15.8
**TP53**	17: 7577121	c.817C>T	p.R273C	Missense	10.4
**ARID1A**	1: 27094421	c.3129dupT	p.V1044Cfs*3	Frameshift	8.5

The patient was discussed in our multi-disciplinary conference and the recommendation was to initiate an immune checkpoint inhibitor (ICI) considering the tumor’s squamous histology. He received the first dose of pembrolizumab in May 2023. In July 2023, the patient was hospitalized for pancreatitis: investigational studies were unremarkable for common etiologies, hence it was likely an immune-related adverse event from the pembrolizumab. Symptoms resolved shortly after admission and steroids were not administered, but pembrolizumab was held for one month until lipase levels normalized and his activity level improved to his baseline of ECOG performance status of 1. A subsequent CT scan in July 2023 demonstrated a significant reduction in both hepatic masses, with the right lobe mass measuring 5.7 cm, decreased from 10.2 cm three months prior, representing an approximate 88% reduction in tumor volume (Figure 3). He has since resumed pembrolizumab and completed six cycles at our institution at the time of last follow-up in September 2023, after which returned to his prior institution to continue therapy.

## DISCUSSION

In the past twenty years, the only major advancement in PDAC treatment has been the transition from a single cytotoxic agent to multiple cytotoxic agents, responsible for an increase in 5-year survival rate of 5% in 1998 to 12% in 2018, still the lowest of all cancers [1]. For patients with metastatic PDAC, the initial standard therapies are either FOLFIRINOX or gemcitabine and albumin-bound paclitaxel [3, 4]. These therapies, however, come at the expense of significant toxicities that can limit their use to the most fit patients [5]. Importantly, the clinical trials of cytotoxic therapies, and the vast majority of clinical trials in pancreatic cancer, exclude adenosquamous and other rare subsets of pancreatic cancer.

There has been an effort to develop targeted therapies for advanced PDAC, albeit with limited success. Olaparib, a polyadenosine diphosphate-ribose polymerase (PARP) inhibitor, was approved for use as maintenance therapy in patients with advanced PDAC harboring germline *BRCA1*/*BRCA2* mutations [6]. However, its use is limited, as *BRCA* germline alterations are only present in approximately 2% of PDAC [7] .

Another target of interest in PDAC is the Kirsten rat sarcoma viral oncogene (*KRAS*) mutation, known to be a major oncogenic driver in 90% of PDAC. The predominant *KRAS* mutation, G12D, constitutes 45% of all *KRAS* mutations in PDAC but remains difficult to target, as do other common *KRAS* mutations. However, recent phase 2 trials of sotorasib and adagrasib, inhibitors of the *KRAS* G12C mutation, demonstrated a 6.9 and 8.0-month median OS, respectively, in patients with advanced previously-treated PDAC [8]. Although these inhibitors have reinvigorated efforts at targeting other *KRAS* mutations, the G12C mutation represents only 1–2% of all *KRAS* mutations in PDAC and is therefore only beneficial to a small proportion of advanced PDAC patients [9, 10].

Immunotherapy has revolutionized cancer therapeutics and ICI are now approved for all solid tumors with high MSI (MSI-H) or deficient MMR (dMMR), including advanced PDAC. However, early clinical phase trials of ICI in advanced PDAC have not been promising. This was illustrated in KEYNOTE-028 and KEYNOTE-158, two trials in which patients with a range of solid cancers, including 22 and 24 PDAC patients, respectively, were treated with pembrolizumab. Importantly, in KEYNOTE-028, all tumors included were PD-L1 positive, while in KEYNOTE-158, they exhibited either MSI-H or dMMR. Amongst PDAC patients, both trials reported a PFS of 2.1, an OS of 3.7 to 4 months and the lowest overall response rates (ORR) of all solid cancers included in the cohorts [11, 12].

The lack of success of ICI in PDAC, as opposed to their effectiveness in other solid tumors, can partially be attributed to the distinctive tumor microenvironment, characterized by a dense desmoplastic stroma, limited vascularity and elevated levels of immunosuppressive cytokines [13]. Additionally, only 1% of PDAC are MSI-H, dMMR or TMB-high. There are, however, a few published cases of patients with advanced PDAC with progression on one or more lines of therapy who subsequently had partial or complete response to ICI. All the patients reported had either high PD-L1 expression or dMMR [14–16]. In contrast, our patient had intact MMR. Unfortunately, PD-L1 status could not be determined because the pathology slides were reviewed at our institution as a second opinion, and no additional tissue was available for PD-L1 staining.

Herein we report a case of an ASCP with intact MMR and an unknown PD-L1 status that showed a significant response to an ICI. Definitionally, ASCP requires ≥30% of the carcinoma to demonstrate squamous differentiation, typically at the periphery and characterized by keratinization and p40 immunostaining [2]. Due to the peripheral localization and sampling bias on biopsies, it is probable that the actual occurrence rate is higher than the 1–5% reported in the literature [17].

There are no prospective studies selectively targeting ASCP, and these patients are typically treated with chemotherapy regimens similar to PDAC, though with worse outcomes [2]. Interestingly, a recent case series demonstrated PD-L1 expression in 5/6 (83%) cases of surgically resected ASCP compared to 1/34 (3%) cases of resected PDAC. All 5 demonstrated PD-L1 expression selectively within the squamous component of the tumor [18]. These results align with those reported in a study of adenosquamous lung carcinomas, where PD-L1 expression was 11% within the glandular component compared to 39% within the squamous component [19]. Furthermore, the tumor microenvironment of lung squamous cell carcinoma has more tumor-infiltrating lymphocytes than lung adenocarcinoma [20]. Therefore, it is plausible that the tumor microenvironment of ASCP is drastically different than that of PDAC, that it drove the excellent response observed with pembrolizumab in our patient and that it may be a promising therapy for ASCP. This highlights the need for studies to evaluate ICI selectively in patients with ASCP. To that point, there is an active multi-center phase 2 trial investigating outcomes and responses to ICI in patients with metastatic or unresectable ASCP or ampullary cancer [19].
